# Overview of programmed electrical stimulation to assess atrial fibrillation susceptibility in mice

**DOI:** 10.3389/fphys.2023.1149023

**Published:** 2023-04-11

**Authors:** Matthew B. Murphy, Prince J. Kannankeril, Katherine T. Murray

**Affiliations:** Departments of Medicine, Pediatrics, and Pharmacology, Vanderbilt University School of Medicine, Nashville, TN, United States

**Keywords:** atrial pacing, intracardiac, transesophageal, mice, atrial fibrillation

## Abstract

Atrial fibrillation (AF) is the most common human arrhythmia and is associated with increased risk of stroke, dementia, heart failure, and death. Among several animal models that have been used to investigate the molecular determinants of AF, mouse models have become the most prevalent due to low cost, ease of genetic manipulation, and similarity to human disease. Programmed electrical stimulation (PES) using intracardiac or transesophageal atrial pacing is used to induce AF as most mouse models do not develop spontaneous AF. However, there is a lack of standardized methodology resulting in numerous PES protocols in the literature that differ with respect to multiple parameters, including pacing protocol and duration, stimulus amplitude, pulse width, and even the definition of AF. Given this complexity, the selection of the appropriate atrial pacing protocol for a specific model has been arbitrary. Herein we review the development of intracardiac and transesophageal PES, including commonly used protocols, selected experimental models, and advantages and disadvantages of both techniques. We also emphasize detection of artifactual AF induction due to unintended parasympathetic stimulation, which should be excluded from results. We recommend that the optimal pacing protocol to elicit an AF phenotype should be individualized to the specific model of genetic or acquired risk factors, with an analysis using several definitions of AF as an endpoint.

## Introduction

Afflicting >37 million people worldwide, atrial fibrillation (AF) is the most common sustained arrhythmia in the Western world (Lippi et al., 2021). AF increases the risk of stroke, dementia, heart failure, and death, including sudden cardiac death (Staerk et al., 2017). Unfortunately, existing therapies for the prevention and treatment of AF are suboptimal due to high recurrence rates and serious associated adverse events (Gupta et al., 2013; January et al., 2014). In order to develop novel AF therapies, animal models have been employed to investigate the molecular determinants of the AF substrate.

**FIGURE 1:**
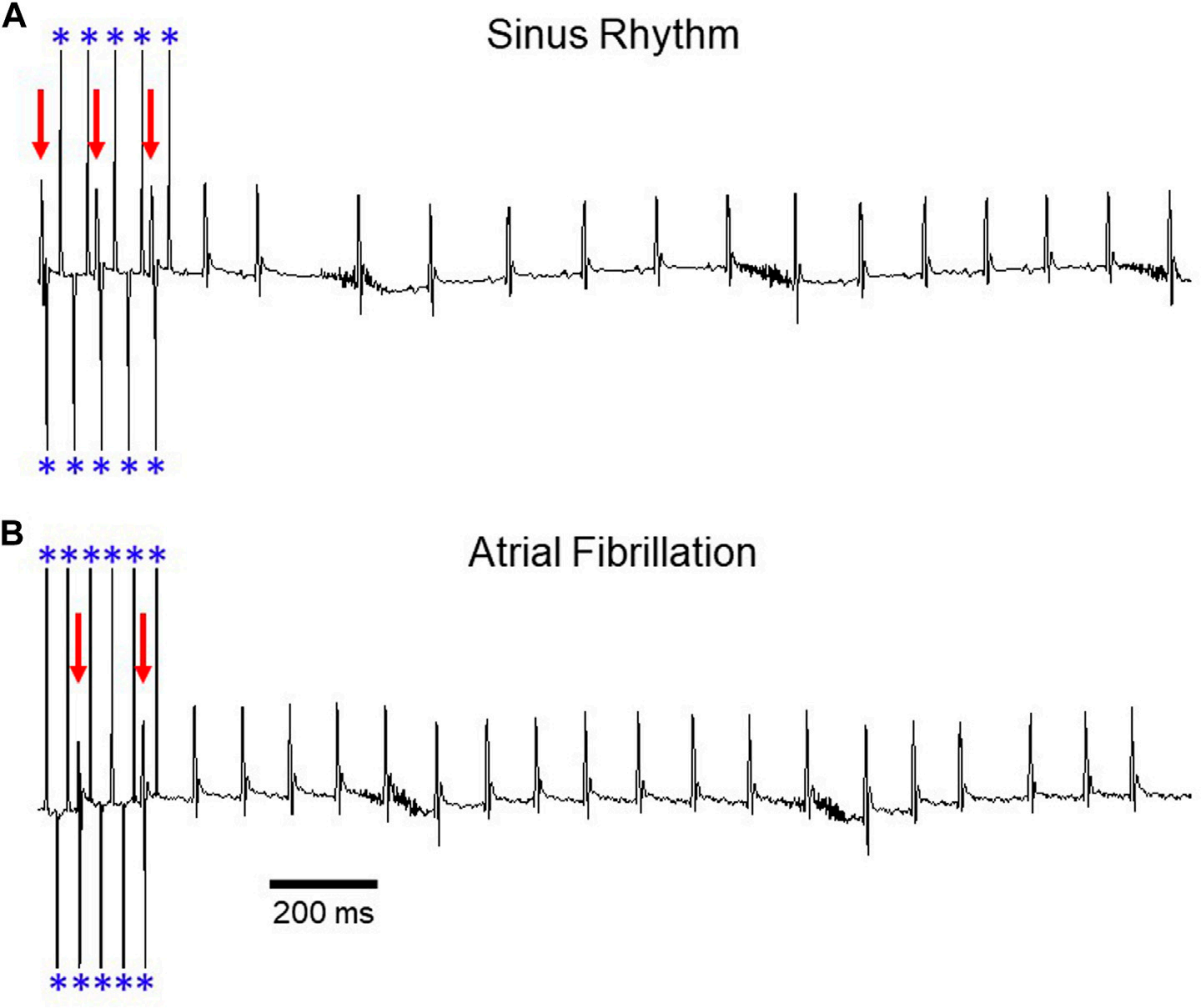
Induction of AF during transesophageal atrial pacing. Surface ECG recordings depicting **(A)** sinus rhythm and **(B)** atrial fibrillation after rapid atrial pacing. In panel **A**, pacing demonstrates 2:1 AV conduction (rate exceeds Wenckebach cycle length), with variable A:V conduction in panel **B**. Red arrows denote QRS complexes and blue asterisks denote atrial pacing spikes. The baseline artifact is related to mouse respiration. Adapted from Murphy MB, Kim K, Kannankeril PJ, Murray KT. Optimization of Transesophageal Atrial Pacing to Assess Atrial Fibrillation Susceptibility in Mice. *J Vis Exp*. (184), e64168, doi:10.3791/64168 (2022).

**FIGURE 2:**
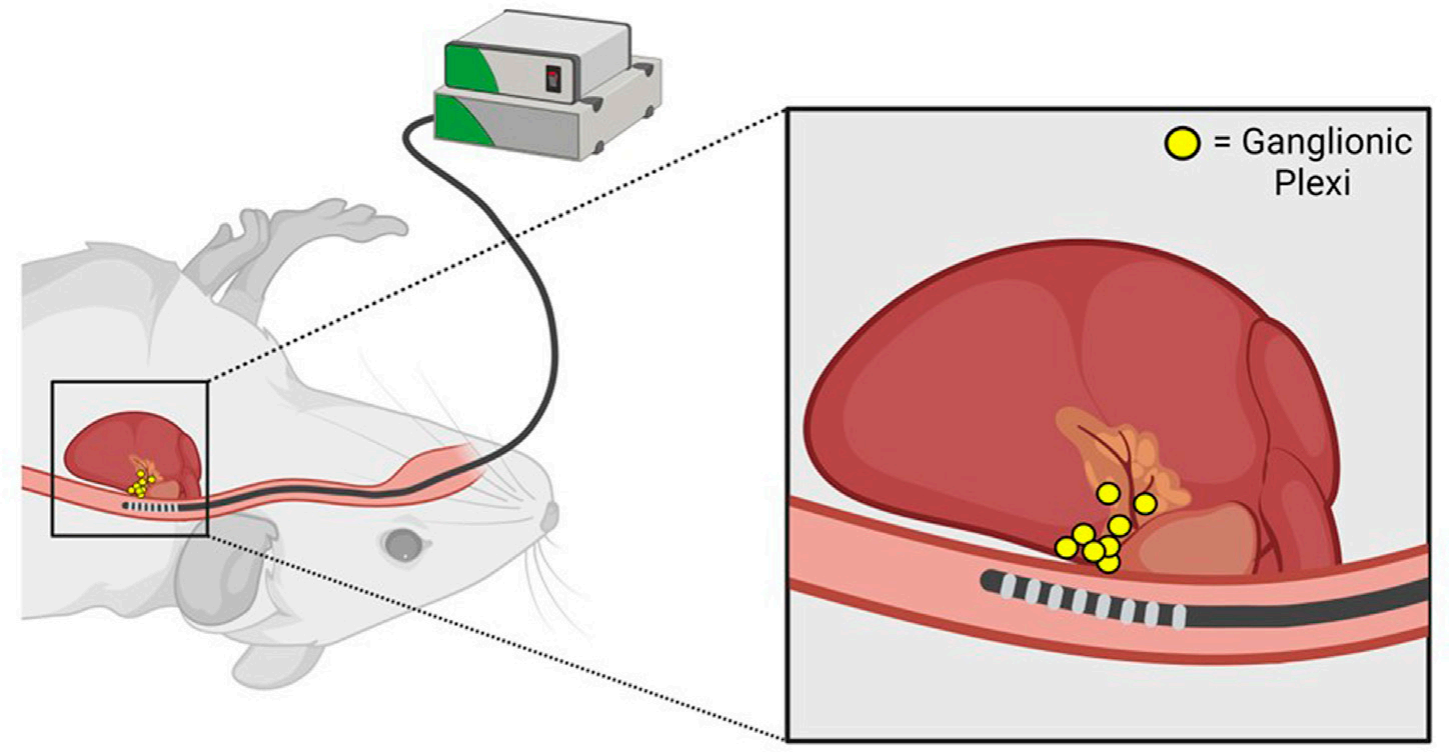
Anatomic basis for parasympathetic stimulation during transesophageal atrial pacing. Proximity of the transesophageal pacing catheter to posterior left atrial ganglionic plexi is illustrated. Adapted from Murphy MB, Kim K, Kannankeril PJ, Murray KT. Optimization of Transesophageal Atrial Pacing to Assess Atrial Fibrillation Susceptibility in Mice. *J Vis Exp*. (184), e64168, doi:10.3791/64168 (2022).

**FIGURE 3:**
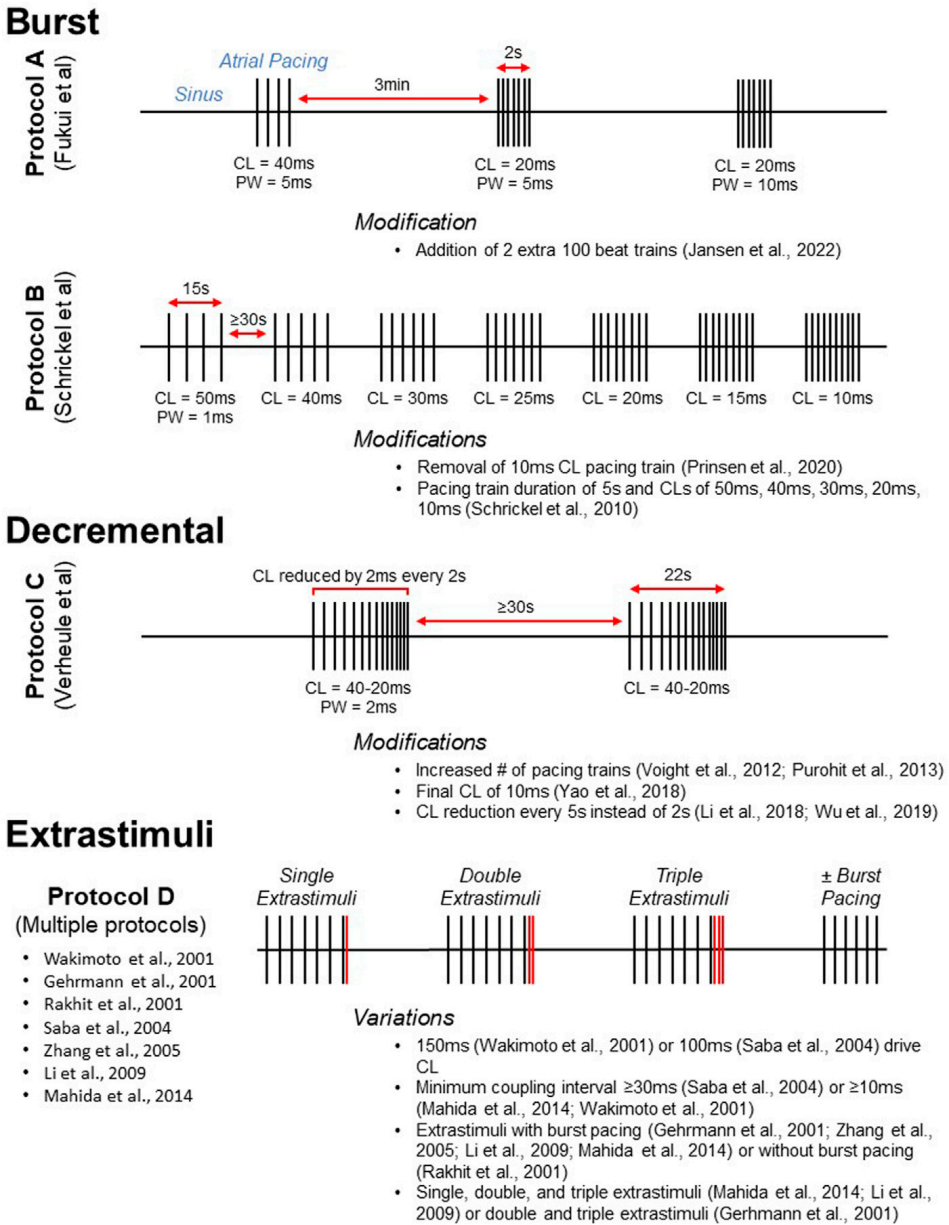
Visual representation of commonly used atrial pacing protocols. Protocols A, B, and C display the timing of stimulus delivery for three widely used burst (Schrickel et al., 2002; Fukui et al., 2013) and decremental (Verheule et al., 2004) atrial pacing protocols. Selected modifications are listed below. For conceptualization, Protocol D (multiple reported protocols) illustrates the delivery of extrastimuli after a pacing train. Protocols utilizing extrastimuli are often, but not always, accompanied by burst pacing. CL, cycle length; PW, pulse width.

Large animals including dogs (Gerstenfeld et al., 2011), goats (Wijffels et al., 1995), and sheep (Anne et al., 2007) have been frequently used to model AF. However, housing costs, limited genetic manipulation, and poor social acceptance have prompted investigation of small animal models instead (Schuttler et al., 2020). While mouse models of AF risk factors are not without limitations (e.g., differences in ion channel expression and shorter action potential durations), they do address a number of limitations encountered with large animals and are often quite similar to human disease (Fukui et al., 2013; Guasch et al., 2013). However, few mouse models develop spontaneous AF (Keefe et al., 2022a) and, as a result, most require programmed electrical stimulation (PES) to assess AF susceptibility (Figure 1). Atrial pacing can be performed by either intracardiac stimulation of the right atrium using a multipolar catheter placed into the heart *via* an internal jugular vein, or by transesophageal pacing, given the close proximity of the esophagus to the posterior left atrium (Figure 2). Several basic pacing modes are used for stimulus delivery including: 1) burst pacing with a constant interstimulus interval, or cycle length (CL); 2) decremental pacing during which the pacing CL becomes progressively shorter (i.e., the rate faster) during the pacing train; and 3) the introduction of premature beats or extrastimuli during sinus rhythm or following a pacing train (Figure 3). Due to a lack of standardized methods, numerous PES protocols have been reported that differ with respect to multiple parameters such as pacing mode and protocol design, stimulus amplitude, and even the definition of AF (Murphy et al., 2022b). Given this complexity, the selection of an appropriate atrial pacing protocol for a specific model has remained arbitrary. Here, we review the development of intracardiac and transesophageal PES, including commonly used pacing protocols, representative experimental models, and advantages and disadvantages of both techniques. Rather than an exhaustive review of all published studies, we have focused on frequently employed protocols described in sufficient detail that they can be easily reproduced, and examples of their modification. We then highlight studies using transesophageal PES that emphasize the development of reproducible pacing parameters for a specific model under study, as well as the detection of artifactual AF induction due to unintended parasympathetic stimulation.

## Intracardiac PES

### Development

Soon after AF was detected in the mouse (Wang et al., 1997; Sah et al., 1999), Wakimoto et al. (2001) used intracardiac PES for induction of AF. It was observed that a parasympathetic agonist, carbamylcholine, increased AF susceptibility in wild-type C57BL/6 mice subjected to atrial burst pacing. Moreover, the authors demonstrated that intracardiac stimulation could be used to determine electrophysiologic parameters including sinus node function (assessed by sinus node recovery time) and the effective refractory period (ERP, or non-conducting time period following a premature beat) of the atrium, atrioventricular (AV) node, and ventricle. These findings prompted the rapid development and widespread use of intracardiac PES in mouse models of AF risk factors (Gehrmann et al., 2001; Rakhit et al., 2001).

### Common protocols

Multiple intracardiac atrial pacing protocols have been reported (Table 1). Despite differences in protocol parameters, many studies use a decremental mode of stimulus delivery as developed by Verheule and coworkers (Figure 3, Protocol C). In the initial report, mice were subjected to two pacing trains with an initial pacing CL of 40 ms that was reduced by 2 ms every 2 s until termination at 20 ms (Verheule et al., 2004). Adaptations of this protocol have included an increased number of pacing trains (Voight et al., 2012; Purohit et al., 2013) as well as a final CL of 10 ms (Yao et al., 2018). Intracardiac PES can also be performed using burst pacing at a fixed CL, and several burst pacing protocols have been reported (Wakimoto et al., 2001). However, the methods developed by Fukui and coworkers for transesophageal PES are most commonly used (Fukui et al., 2013; Egom et al., 2015; Fukui et al., 2017; Jansen et al., 2019). In this protocol, pacing was delivered in three trains that were 2s in duration (Figure 3, Protocol A). The initial 2 s burst had a CL of 40 ms and a pulse width of 5 ms while the second and third bursts had CLs of 20 ms with pulse widths of 5 and 10 ms, respectively. A subsequent modification of this protocol was the addition of two 100 beat trains delivered at 20–25 ms pulse widths after the third 2 s burst (Jansen et al., 2022).

**TABLE 1:** Selected studies utilizing intracardiac PES to induce AF.

References	Intervention/Model	Pacing mode	Amplitude (mA)	Definition of AF		
				Episode	Regularity	Susceptibility
Wakimoto et al. (2001)	Parasympathetic stimulation	Extrastimuli, burst	NR	NR	Reg/Irreg	Incidence, duration
Gehrmann et al. (2001)	Myocardial infarction	Extrastimuli, burst	NR	NR	NR	Incidence
Rakhit et al. (2001)	eNOS deficiency	Extrastimuli, burst	NR	NR	NR	Incidence
Sood et al. (2008)	FKBP12.6 deficiency	Decremental	1.5x TH	≥1s	Irreg	Incidence, duration
Voight et al. (2012)	RyR2 S2814D knock-in	Decremental	1.5x TH	≥1s	NR	Incidence
Purohit et al. (2013)	Angiotensin II infusion	Decremental	NR	≥1s	Irreg	Incidence
Yao et al. (2018)	Constitutively active NLRP3	Decremental	1.5x TH	≥1s	Irreg	Incidence
Egom et al. (2008)	NPR-C deficiency	Burst	0.4 mA	≥1s	Irreg	Incidence, duration
Jansen et al. (2019)	Angiotensin II infusion, NPR-C deficiency	Burst	NR	≥1s	Irreg	Incidence, duration
Scott et al. (2021)	Obesity, NLRP3 deficiency	Decremental	NR	≥2s	NR	Incidence, duration
Jansen et al. (2017)	Aging	Burst	NR	≥1s	Irreg	Incidence, duration
Saba et al. (2005)	TNF-α overexpression	Extrastimuli, burst	2x TH	10 beats	NR	Incidence
Li et al. (2018)	Angiotensin II infusion, PSMB10 deficiency	Burst	NR	≥1s	Irreg	Incidence, duration
Wu et al. (2019)	Angiotensin II infusion	Burst	NR	NR	NR	Incidence, duration
Polina et al. (2020)	Akita - type 1 diabetes	Burst	NR	≥1s	Irreg	Incidence, duration
Bohne et al. (2021)	db/db - type 2 diabetes	Burst	NR	≥1s	Irreg	Incidence, duration
Jin et al. (2019)	Streptozotocin injection - type 1 diabetes	Burst	NR	≥1s	Irreg	Incidence, duration, episode
Wang et al. (2018)	Abdominal aortic constriction, ALK4 deficiency	Decremental	NR	≥1s	Irreg	Incidence, Duration
Campbell et al. (2020)	SPEG deficiency	Decremental	NR	>1s	NR	Incidence
Bapat et al. (2022)	Genetic inhibition of serum glucocorticoid kinase 1	Extrastimuli, burst	NR	>1s	NR	Incidence, duration, episode

Regularity refers to atrial signal. Reg, regular; Irreg, irregular; NR, not reported; TH, diastolic threshold; eNOS, endothelial nitric oxide synthase; RyR2, ryanodine receptor two; NLRP3, NOD-, LRR-, and pyrin domain-containing protein three; TNF-α, tumor necrosis factor alpha; PSMB10, proteasome 20S subunit beta 10; ALK4, activin receptor-like kinase 4; SPEG, striated muscle preferentially expressed protein kinase.

### Examples of use

Intracardiac PES is routinely used in mouse models of AF risk factors such as inflammation, hypertension, and genetic variants. Using a decremental pacing protocol, Yao et al. (2018) found that activation of the cardiomyocyte NLRP3 (NACHT, LRR, and PYD domain containing protein 3) inflammasome increased AF susceptibility in mice. In this study, AF was considered to be an endpoint if two of the three pacing trains induced the arrhythmia. While some studies required similar reproducibility for a positive finding (Scott Jr et al., 2021), others reported all episodes of inducible AF (Jansen et al., 2017). It was reported that cardiomyocyte overexpression of tumor necrosis factor alpha (*Tnfa*) increased AF vulnerability after atrial burst pacing at CLs of 100–50 ms. However, pacing train duration was not reported limiting future applications of this protocol (Saba et al., 2005).

Angiotensin II (Ang II) infusion is a well-established mouse model of hypertension-mediated AF (Schluttler et al., 2020), and a variety of intracardiac atrial pacing protocols have been reported to elicit AF in these mice. One study determined that the decremental protocol initially described by Verheule could induce AF after a 3 weeks infusion of Ang II (Purohit et al., 2013). Others have modified the protocol in this model, including a reduction of the pacing CL by 2 ms every 5 s instead of every 2 s (Li et al., 2018; Wu et al., 2019). Using an atrial burst pacing protocol (Fukui et al., 2013; Fukui et al., 2017), it was demonstrated that activation of natriuretic peptide receptor-C (NPRC) protects against hypertension-mediated AF (Jansen et al., 2019). In addition to AF incidence, this study also characterized AF episodes as brief (<5 s), non-sustained (5–30 s), or sustained (>30 s). Using this classification, only sustained AF episodes were increased in Ang II-treated mice compared to controls, indicating the utility of a severity analysis in at least some AF models.

Multiple other AF risk factors have been modeled in mice, including diabetes and cardiomyopathy. Atrial burst pacing using the Fukui method demonstrated that loss of insulin signaling increased AF incidence and duration in type 1 diabetic Akita mice (Polina et al., 2020), as well as AF induction in type 2 diabetic db/db mice (Bohne et al., 2021). Another study reported that decremental pacing increased AF susceptibility in streptozocin-induced diabetes (Jin et al., 2019). Pacing was performed using a modified version of the Verheule protocol with CL reductions occurring every 5 s. In a mouse model of cardiomyopathy, Wang et al. found that haploinsufficiency of activin receptor-like kinase 4 (*Acvr1b*) reduced AF vulnerability. The authors also used an adaptation of the Verheule method with initial and final CLs of 50 and 10 ms, respectively (Wang et al., 2018). A series of burst pacing trains was used to determine that mice deficient in desmin (*Des*) were susceptible to AF and ventricular tachycardia (Schrickel et al., 2010). Atrial arrhythmias were induced by pacing in 5s intervals at CLs of 50, 40, 30, 20, and 10 ms, with the protocol performed initially with a stimulus amplitude of 1 mA, which was repeated using 2 mA.

In addition to ion channel mutations, genetic variants linked to AF have been studied in mice, including altered expression of genes encoding the paired-like homeodomain transcription factor 2 (*Pitx2*) and the potassium calcium-activated channel subfamily N member 3 (*Kcnn3*; Schuttler et al., 2020). Mice deficient in *Pitx2* were initially found to be susceptible to AF by decremental pacing using the Verheule protocol (Wang et al., 2010). Using both burst pacing as well as PES with extrastimuli, it was found that overexpression of *Kcnn3* resulted in atrial arrhythmias (Mahida et al., 2014; Figure 3, Protocol D). Burst pacing was performed at CLs of 50 or 30 ms for up to 1 min of stimulation. In addition, single, double, and triple extrastimuli were introduced following a drive CL of 100 ms, with a minimum coupling interval of 10 ms. This protocol has been used to induce AF in murine models by other investigators as well (Zhang et al., 2005; Li et al., 2009). Additional recent studies are included in Table 1 and Table 2 that represent further modifications of the studies illustrated in Figure 3 for both intracardiac and transesophageal pacing.

**TABLE 2:** Selected studies using transesophageal PES to induce AF.

References	Intervention/Model	Pacing mode	Amplitude (mA)	Definition of AF		
				Episode	Regularity	Susceptibility
Hagendorff et al. (1999)	Connexin 40 deficiency	Burst	NR	NR	Reg/Irreg	Observation
Schrickel et al. (2002)	C57Bl/6 wild-type	Burst	1–4 mA	≥1s	Irreg	Incidence, duration
			2x TH			
Prinsen et al., 2020	Angiotensin II infusion	Burst	3 mA	≥1s	NR	Duration
Suita et al. (2020)	Occlusal disharmony	Burst	1.5 mA	≥2s	NR	Duration
Faggioni et al. (2014)	Calsequestrin deficiency	Burst	2x TH	≥0.15s	Irreg	Episode, duration
Suffee et al. (2022)	High-fat diet	Burst	NR	≥0s	NR	Incidence, duration
Fukui et al. (2017)	High-fat diet, leptin deficiency	Burst	NR	≥1s	Irreg	Incidence, duration
Sato et al. (2019)	Perilipin 2 overexpression	Burst	NR	≥5min	Irreg	Sustained AF
Aschar-Sobbi et al. (2015)	Endurance exercise	Decremental, burst	1.5x TH	≥10s	Irreg	Incidence, duration
Maria et al. (2020)	Insulin deficiency	Burst	NR	≥0s	NR	Incidence, duration
Verheule et al. (2004)	TGF-β1 overexpression	Decremental	1.5x TH	≥2s	Irreg	Incidence, duration
Zhan et al. (2020)	Angiotensin II infusion	Burst	NR	≥1s	Irreg	Incidence, duration
Xie et al. (2015)	RyR2-R2474S and RyR2-S2808D knock-in	Decremental	NR	≥1s	Irreg	Incidence
Fossier et al. (2022)	Metabolic syndrome	Burst	1 mA	≥1s	Irreg	Incidence
Mighiu et al. (2021)	NOX2 overexpression	Decremental	2x TH	≥2s	Irreg	Incidence, duration
Menon et al. (2019)	Frameshift *NPPA* mutation	Burst, decremental, other	NR	≥5s	Irreg	Episode, incidence, duration
Bosada et al. (2023)	TBX5 mutants	Decremental	2x TH	>1s	NR	Incidence, duration
Lai et al. (2022)	Cardiac-specific transgenic TGF-β	Burst	1.5x TH	>3s	Irreg	Incidence, duration
Gong et al. (2022)	Chronic pain	Decremental	NR	≥2s	Irreg	Incidence

Regularity refers to atrial signal. Reg, regular; Irreg, irregular; NR, not reported; TH, diastolic threshold; TGF-β, transforming growth factor beta; RyR2, ryanodine receptor two; NOX2, NADPH, oxidase two; NPPA, atrial natriuretic peptide gene; TBX5, T-box transcription factor 5.

### Advantages and disadvantages

One advantage of intracardiac PES is the ability to record a His potential, signifying the onset of ventricular conduction. This enables measurement of the AH interval, which is conduction from the right atrium to the His bundle and largely reflects AV nodal conduction, and the HV interval, representing conduction from the His bundle to ventricular myocardium. In addition, one can more precisely determine the atrial and AV nodal ERPs. During transesophageal pacing, stimulus artifacts are large and often obscure atrial signals (Etzion et al., 2008) which, in turn, prevents distinguishing between atrial capture with block in the AV node (AVNERP) or if atrial capture was lost (AERP). In contrast, stimulus artifacts are minimal during intracardiac PES so that atrial capture can generally be determined (Li and Wehrens, 2010; Hennis et al., 2022). Additionally, because a His potential is recorded, the AV nodal ERP can also be accurately determined. Another advantage of intracardiac PES is the ability to assess ventricular arrhythmia vulnerability (Hennis et al., 2022). However, this advantage may no longer be valid as recent reports indicate that the transesophageal approach can be used to induce ventricular tachyarrhythmias (Kim et al., 2021; Schmeckpeper et al., 2021). There are multiple disadvantages to intracardiac PES including the amount of time required to study an individual mouse. Studies may last up to 2 h (Li and Wehrens, 2010) which can increase the risk of anesthetic influence on electrophysiologic parameters. Moreover, the required cardiac instrumentation is technically challenging and requires extensive training to avoid procedural errors such as excessive bleeding (Li and Wehrens, 2010). In addition, intracardiac PES is a terminal procedure in the mouse.

## Transesophageal PES

### Development

Hagendorff and coworkers were the first to use transesophageal PES in mice. Utilizing a burst protocol, the authors found that mice lacking connexin 40 (GJA5) were highly susceptible to atrial arrhythmias (Hagendorff et al., 1999). While AF incidence was reported, additional information including total AF duration and episode number was not described. Subsequently, a PES method was reported for AF induction in wild-type C57Bl/6 mice (Schrickel et al., 2002). This work demonstrated the importance of stimulus strength in promoting atrial arrhythmias, as induction of AF in wild-type mice was minimal at low stimulus amplitudes. Ultimately, these findings resulted in more widespread use of transesophageal PES for AF induction in mouse models.

### Common protocols

As with intracardiac PES, transesophageal atrial pacing protocols have primarily used either a burst or decremental mode of stimulus delivery (Table 2). In a novel method, burst pacing was performed in sequential 15 s intervals at CLs of 50, 40, 30, 25, 20, 15, and 10 ms (Schrickel et al., 2002; Figure 3, Protocol B). While this initial study only included wild-type C57Bl/6 mice, the protocol was subsequently employed to demonstrate increased vulnerability to atrial arrhythmias in a mouse model of calsequestrin (*Casq2*) deficiency (Faggioni et al., 2014). A modification of this method was removal of the 10 ms CL pacing train (Prinsen et al., 2020). Additional transesophageal burst pacing methods have been reported (Hagendorff et al., 1999; Fukui et al., 2013; Fukui et al., 2017). However, they were either not widely adopted for this mode of pacing (Hagendorff et al., 1999), or they have been primarily used for intracardiac PES (Fukui et al., 2013; Fukui et al., 2017). The most common decremental pacing protocol for transesophageal PES was that developed by Verheule and others described above, in which mice are subjected to two pacing trains with a gradual reduction in the pacing CL (Verheule et al., 2004).

### Examples of use

Multiple metabolic disorders are linked to AF, including obesity as well as diabetes mellitus. Mice on a high-fat diet demonstrated increased AF vulnerability in response to transesophageal pacing using a single 3 s burst due to electrical and metabolic remodeling (Suffee et al., 2022; additional details such as pulse width not reported). Using their own method, Fukui and others demonstrated that hyperleptinemia increased AF susceptibility in mice maintained on a high-fat diet (Fukui et al., 2017). Another study reported that perilipin 2 (*Plin2*) overexpression increased sustained AF in mice due to atrial steatosis (Sato et al., 2019). While these authors defined sustained AF as an episode lasting longer than 5 min, other definitions have been more commonly used, including 10 s (Aschar-Sobbi et al., 2015), 15 s (Bruegmann et al., 2018), and 30 s (Jansen et al., 2017; Jansen et al., 2019). Transesophageal PES has also been used to induce AF in a mouse model of Type 1 diabetes (Maria et al., 2020).

As with intracardiac PES, several transesophageal atrial pacing protocols have been shown to induce AF in murine models of hypertension using chronic Ang II infusion, including the Fukui protocol (Fukui et al., 2013; Zhan et al., 2020). Reactive mediators of oxidative stress known as isolevuglandins were found to be drivers of AF in Ang II-infused hypertensive mice using a modification of the Schrickel protocol (Prinsen et al., 2020).

Using transesophageal PES, several studies have investigated the role of mitochondrial dysfunction and oxidative stress in AF pathogenesis. It was reported that mitochondrial oxidative stress drove AF due to oxidation of the type 2 ryanodine receptor (RYR2; Xie et al., 2015). The Verheule protocol was employed for AF induction, although the final pacing CL was lowered from 20 to 10 ms. Protocol details including the number of pacing trains delivered, pulse width, and stimulus amplitude were not described, hindering future use of this protocol. Another investigation attributed AF susceptibility during the metabolic syndrome to decreased mitochondrial calcium uptake (Fossier et al., 2022). Burst pacing trains were 30 s in duration, with an initial CL 30 ms shorter than the sinus RR interval. For subsequent pacing trains, the CL was reduced by 10 ms until termination at 30 ms. Overexpression of *NOX2* which encodes NADPH oxidase 2, a major source of reactive oxygen species, promoted inducibility of AF, but not its stability (Mighiu et al., 2021). This study employed an adaptation of the Verheule protocol, with atrial pacing initiated at a CL of 60 ms and subsequently reduced by 2 ms every 2 s until termination at 10 ms.

Transesophageal PES has also been successfully employed to investigate the molecular mechanisms of genetic causes of AF. Using a unique combination of burst and decremental pacing, it was determined that a frameshift mutation in the *NPPA* gene encoding natriuretic peptide precursor A increased AF vulnerability in mice due to electrical remodeling (Menon et al., 2019). Pacing was performed in sequential bursts of 300 cycles at CLs of 50, 40, 30, 25, 20, and 15 ms, followed by the Verheule decremental protocol. Mice were then subjected to burst pacing with 12 trains that were either 50 or 30 ms in length. As noted above, a modified version of the Schrickel method was used to demonstrate that loss of *Casq2* promoted murine AF susceptibility due to spontaneous diastolic Ca^2+^ elevations (Faggioni et al., 2014). Murphy and coworkers demonstrated that *Pitx2*-deficient mice were susceptible to AF using both burst and decremental transesophageal atrial pacing modes, suggesting that multiple methods can be useful to assess AF susceptibility in some models (Murphy et al., 2022b).

### Advantages and disadvantages

A major advantage of transesophageal PES is the ability to perform repeated testing in the same animal (Schrickel et al., 2002). Unlike intracardiac PES, transesophageal atrial pacing is a survival procedure, allowing individual mice to be restudied over time or with different pacing protocols (Murphy et al., 2022a). Another advantage of transesophageal PES is a short study duration (∼20 min). As described earlier, intracardiac pacing can last several hours which not only reduces the number of mice that can be studied at one time, but also increases the risk of confounding anesthetic effects (Constantinides et al., 2011). The disadvantages of transesophageal pacing include an inability to record the His potential, to accurately measure atrial and AV nodal ERP and, in some cases, to accomplish reliable ventricular stimulation (Hennis et al., 2022). Finally, excessive parasympathetic stimulation causing AV block during pacing may occur which can confound results (see below; Murphy et al., 2022b).

## Protocol optimization for transesophageal PES

When using transesophageal PES, several factors can influence AF inducibility, including age, sex, and the pacing protocol employed. AF susceptibility increases as mice age (Luo et al., 2013; Jansen et al., 2017), and determining an age window when AF is inducible in the model under study but not in control mice is essential (Keefe et al., 2022b). In addition, only one sex may demonstrate an AF phenotype (Keefe et al., 2022b; Murphy et al., 2022b). Finally, we recently showed that for optimal reproducibility, pacing mode and parameters should be optimized in pilot studies for the specific model under investigation (Murphy et al., 2022b). For example, we found that *Pitx2*-deficient mice displayed AF inducibility using both burst and decremental pacing, whereas only burst pacing provoked AF in mice with systemic inflammation (Murphy et al., 2022b). In this investigation, male and female mice were subjected to decremental and burst pacing every other week beginning at 8 weeks of age to identify the ideal age, sex, and pacing mode for subsequent studies. Using this approach, the optimal pacing conditions to elicit an AF phenotype in models of genetic and acquired risk factors were identified (Murphy et al., 2022a).

Another source of variability is the definition of AF as an endpoint (Table 2). The majority of studies define an AF episode as 1 s or more of rapid atrial activity with an irregularly irregular response (Voight et al., 2012; Xie et al., 2015; Fukui et al., 2017). However, AF susceptibility as an endpoint could potentially be defined as AF incidence (Fossier et al., 2022), total AF duration (Prinsen et al., 2020), sustained AF incidence (Jansen et al., 2019), and/or the number of AF episodes per mouse (Jin et al., 2019). Pilot studies can determine the number of pacing trains (typically at least 3) during decremental pacing that are optimal for AF detection, and whether short CLs below 20 ms during either decremental or burst pacing should be excluded due to excessive parasympathetic stimulation (see below). Depending upon the model studied, one or more specific definitions of AF susceptibility may reveal an AF phenotype (Mighiu et al., 2021; Murphy et al., 2022a). Therefore, it is essential to analyze AF susceptibility in multiple ways.

During transesophageal PES, inadvertent parasympathetic stimulation can occur due to pacing-induced excitation of ganglionic plexi on the posterior left atrium (Figure 2). This phenomenon is manifested by an excessive increase in the RR interval during pacing (as quantified in Murphy et al., 2022b), indicating the development of prominent slowing of AV nodal conduction and AV block, that is, often associated with artifactual AF induction in control mice. Notably, pacing-induced AV block can be minimized by using a stimulus amplitude ≤ twice diastolic threshold which should be optimized by careful catheter positioning (Murphy et al., 2022a), as well as longer pacing CLs. However, a subset of mice will inevitably experience parasympathetically-mediated AF induction. These animals should be excluded from analysis to increase specificity and facilitate reproducibility between studies.

## Limitations of PES

Despite the widespread use of PES in mice, several challenges and limitations persist. A major issue is the frequency with which critical protocol details for published methods are not reported, including essential pacing parameters (e.g., stimulus intensity, pulse width, *etc.*). This not only prevents reproducibility but may limit the conclusions of the study. Another challenge is a lack of studies that compare PES protocols. Pacing protocols differ for multiple parameters, and it is largely unknown which strategies may be superior or inferior at inducing AF for a specific murine model. For transesophageal PES, we recently demonstrated that AF induction varies depending upon the protocol used (Murphy et al., 2022b) and provided an optimized strategy to develop transesophageal pacing methods. However, to the best of our knowledge no protocol comparisons have been reported for intracardiac PES and such studies would be valuable for future studies. An additional challenge of PES is interpretating the results with reference to the specific protocol used. For example, multiple iterations of the Verheule protocol are reported (Figure 3) with the number of pacing trains varying from 2 (Verheule et al., 2004) to 5 (Purohit et al., 2013). Recently, we demonstrated that an increased number of pacing trains in the Verheule protocol improved the statistical significance between experimental and control mice (Murphy et al., 2022b), suggesting that reproducibility may be enhanced by increasing pacing replicates.

Not infrequently, results obtained using PES do not report whether a regular tachycardia (i.e., atrial flutter or tachycardia) was observed. While a majority of studies define AF as a rapid and irregular atrial rhythm with an irregular ventricular response, many do not delineate regularity (Table 1; Table 2). This can be problematic given that at least in some rodents, atrial tachycardia can lead to prolonged episodes of regular arrythmias that may bias results. Nevertheless, only a handful of studies report regular atrial arrhythmias in mice (Table 1; Table 2), and these responses were most consistent with atrial flutter. Recent studies in rats suggest this limitation may be minimized by 1) quantifying AF with duration scores (Klapper-Goldstein et al., 2020) and 2) using a waveform complexity algorithm to objectively assess arrhythmia regularity (Murninkas et al., 2023).

## Conclusion

Numerous intracardiac and transesophageal atrial pacing protocols have been described to induce AF in mice, with considerable variability in protocol parameters as well as the definition of AF susceptibility between studies. To increase the reproducibility of PES results, pilot studies are useful to optimize protocol design for each model under study.
